# Advances in Photonic Devices Based on Optical Phase-Change Materials

**DOI:** 10.3390/molecules26092813

**Published:** 2021-05-10

**Authors:** Xiaoxiao Wang, Huixin Qi, Xiaoyong Hu, Zixuan Yu, Shaoqi Ding, Zhuochen Du, Qihuang Gong

**Affiliations:** 1 Collaborative Innovation Center of Quantum Matter & Frontiers Science Center for Nano-Optoelectronics, State Key Laboratory for Mesoscopic Physics & Department of Physics, Beijing Academy of Quantum Information Sciences, Peking University, Beijing 100871, China; xiaoxiaowang@stu.pku.edu.cn (X.W.); qihuixin@stu.pku.edu.cn (H.Q.); foxherd@pku.edu.cn (Z.Y.); 1900011367@pku.edu.cn (S.D.); duzhuochen@pku.edu.cn (Z.D.); qhgong@pku.edu.cn (Q.G.); 2 Peking University Yangtze Delta Institute of Optoelectronics, Nantong 226010, China; 3 Collaborative Innovation Center of Extreme Optics, Shanxi University, Taiyuan 030006, China

**Keywords:** phase change materials, photonic devices, reconfigurable, modulator, optical switch, optical logic devices, vanadium dioxide, chalcogenide, photonic neural networks

## Abstract

Phase-change materials (PCMs) are important photonic materials that have the advantages of a rapid and reversible phase change, a great difference in the optical properties between the crystalline and amorphous states, scalability, and nonvolatility. With the constant development in the PCM platform and integration of multiple material platforms, more and more reconfigurable photonic devices and their dynamic regulation have been theoretically proposed and experimentally demonstrated, showing the great potential of PCMs in integrated photonic chips. Here, we review the recent developments in PCMs and discuss their potential for photonic devices. A universal overview of the mechanism of the phase transition and models of PCMs is presented. PCMs have injected new life into on-chip photonic integrated circuits, which generally contain an optical switch, an optical logical gate, and an optical modulator. Photonic neural networks based on PCMs are another interesting application of PCMs. Finally, the future development prospects and problems that need to be solved are discussed. PCMs are likely to have wide applications in future intelligent photonic systems.

## 1. Introduction

Photonic devices and applications have attracted much attention over the last two decades owing to their unique advantages. Photonic devices can achieve the same function as electronic devices using photons rather than electrons. The advantages of photonic devices are their ultrafast information-processing speed owing to the propagation speed of light, very large information capacity because of light’s abundant degrees of freedom, and ability to achieve parallel computing and device interconnection owing to no ohmic loss and Coulomb’s law.

Tremendous progress has recently been made in photonic integrated circuits (PICs), which have the striking advantages of small footprints, low power consumption, ultrahigh information-processing speed, and wide frequency bandwidth. PICs provide a scalable hardware platform to solve the contradiction between the rate of energy efficiency improvement and the rapidly increasing computational load [1]. There are multiple material platforms to achieve PICs. The silicon-on-insulator platform fabrication infrastructure is compatible with complementary metal–oxide–semiconductor (CMOS) technology, and it can significantly reduce production costs. However, electrically pumped efficient sources of silicon are still a challenge because silicon is an indirect bandgap material. To solve this problem, other materials that have a variety of functions, such as InAs, GaAs, LiNbO_3_, and InP, are directly placed on silicon to obtain a heterogeneous silicon PIC platform. The heterogeneous silicon PIC platform provides not only low power and low cost but also high capacity and high volume. Furthermore, some of these platforms can provide modulators with high performance, nonlinearities, and magnetic properties [2].

The functional materials that can achieve proactive control described above completely depend on the external drive. The properties of the materials will be completely or partly restored after removing the external drive, so the response of the optical device will return to the initial state. That is, optical devices based on the materials mentioned above do not have the ability to remember. Electronic integrated circuits have the ability to be redesigned by field-programmable gate arrays, and they provide “programmable” capability based on electronic “rewiring” for various applications. Phase-change materials (PCMs) that have traditionally been used in electrical memory have recently emerged as an ideal material platform to realize optical programmability [3,4]. PCMs offer a way of tuning the properties of the nanostructures that strongly interact with light. Optical property modulation in PCM systems originates from a change in the bonding configuration [5]. There are some phase-change models that correctly describe the mechanism of phase transitions. Density functional theory (DFT) is usually used to predict the phase and electronic structures of PCMs [6]. The local density approximation plus dynamical mean-field theory has recently been proven to be very successful in modeling the symmetry conserving metal–insulator transition of some PCMs, and it is generally used for calculating the electronic spectrum, energy gap, and local magnetic moment [7,8]. The Stefan problem can describe the phenomenon of the phase change by transforming the problem into determining the temperature distribution in the one-dimensional liquid phase at a certain time. It improves the accuracy and stability of the existing solutions of the one-dimensional Stefan problem with time-dependent Dirichlet boundary conditions [9].

PCMs have also attracted much attention for use in reconfigurable photonic devices because of their outstanding properties, such as fast and reversible switching of the states, good thermal stability, drastic nonvolatile changes in the optical properties between the crystalline and amorphous states accompanying considerable modulation in the electrical resistivity, and optical constants with a wide spectral region [10,11]. These excellent properties greatly improve the performance of optical switches, as well as optical modulators in some aspects. Furthermore, phase-change memory offers multilevel data storage and can be applied in both neuro-inspired and all-photonic in-memory computing [12]. The progress in photonic applications focusing on realizing reconfigurable and reprogrammable functions has recently caught up with electronic devices [13]. However, many PCMs, such as Ge–Sb–Te (GST) alloys, simultaneously exhibit large contrast of both the refractive index and optical loss. The concurrent index and loss change fundamentally limit the scope of PCMs in many optical applications [6].

In this review, we provide a general overview of PCMs classified into two types (chalcogenide compounds and metal oxides) and compare the properties of the two types of PCMs. The physical mechanism of the phase transition and the models of PCMs are presented. The role of PCMs in different photonic devices is introduced, ranging from multilevel optical switches and ultracompact and low-loss optical modulators to large-scale photonic deep neural networks. The review emphasizes the important role of PCMs in emerging phase-change photonic devices, such as large-scale photonic deep neural networks and integrated photonic on-chip devices. The main issues facing PCMs and the possible solutions are discussed. The outlook for successfully implementing PCMs in photonic computing is also discussed.

## 2. Phase-Transition Mechanism and Properties of Prototypical PCMs

The most attractive PCMs are chalcogenide compounds (e.g., Ge_2_Sb_2_Te_5_, GeTe, and Ge–Sb–Se–Te) and transition-metal oxides (e.g., VO_2_ and NbO_2_). There are distinct differences between PCMs, such as the volatility, refractive index contrast before and after the phase change, and phase-transition speed [14]. Ge_2_Sb_2_Te_5_ (GST) is a commonly used PCM with fast crystallization speed, large optical and electrical contrast, and good reversibility between the amorphous and crystalline states. The amorphous GST film exhibits semiconductor characteristics, while crystalline GST films exhibit semimetallic and metallic properties in different crystalline states at specific temperatures. The characteristic material of another type of PCM is vanadium dioxide (VO_2_). By increasing the temperature, VO_2_ undergoes an insulator-to-metal transition around its phase-transition temperature, so VO_2_ has substantial optical contrast [15]. Upon decreasing the temperature of VO_2_ below the phase-transition temperature, its state reverts back to the initial structure [16]. As a result, VO_2_ is unsuitable for nonvolatile and reconfigurable applications. Therefore, PCMs can help to realize novel photonic devices and catch up with the requirements for a higher level of integration, adjustability, and performance [4].

Before understanding how PCMs can be applied to optical devices, more needs to be known about the phase-transition procedure itself, both theoretically and experimentally. In this section, we introduce the mechanism of the phase transition, as well as the models and properties of PCMs.

### 2.1. GST and Other Chalcogen-Based Optical PCMs

#### 2.1.1. Models and Physical Mechanism of the Phase Transitions of GST

**Figure 1:**
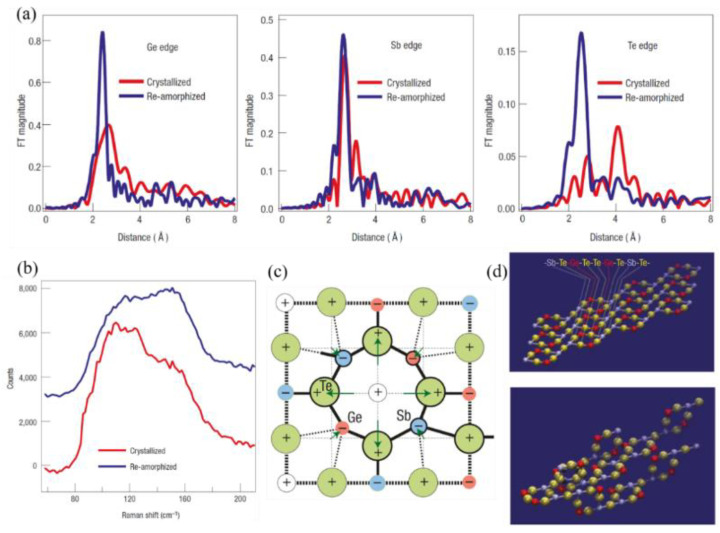
The models and physical mechanism of Ge_2_Sb_2_Te_5_ (GST) phase transitions. (**a**) Fourier-transformed raw extended X-ray absorption fine-structure spectroscopy (EXAFS) spectra for crystallized and laser-amorphized samples. Spectra measured at the K-edges of Ge, Sb, and Te. On amorphization, the bonds become shorter (as shown by shifts in the peak positions) and stronger, that is, more locally ordered (as shown by increases in the peak amplitudes and concurrent decreases in the peak widths). (**b**) Raman scattering spectra for crystallized and re-amorphized GST layers. (**c**) The crystal structure of laser-amorphized GST. A schematic two-dimensional image of the lattice distortion of the rocksalt structure due to charge redistribution between the constituent elements. (**d**) The ideal structure of GST, constructed from the above building blocks and fragments of the three-dimensional GST structure constructed from rigid building blocks.

Kolobov et al. [17] developed a model to explain the mechanism of the phase-transition process of the GST alloy that fits the experimental data well. The distinction between the two states was measured by extended X-ray absorption fine-structure spectroscopy and Raman scattering spectroscopy to construct the model and explain the mechanism. The obtained results (Figure 1a,b) were in good agreement with previous X-ray diffraction measurements.

They determined that the true structure of the crystalline phase is not a typical type of rock-salt structure, although it is similar. The Ge and Sb atoms may swap places, producing randomness, and each additional building block may rotate by 90° in an arbitrary direction. In addition, the ideal model exhibits a stacking sequence of –Sb–Te–Ge–Te–Te–Ge–Te–Sb–Te–, which is identical to the stable hexagonal structure of GST, thus predicting the phase change to some extent. The structure is shown in Figure 1c.

Laser-induced amorphization results in a drastic shortening of covalent bonds of GST (Te–Ge and Te–Sb bonds) and a decrease in the mean-square relative displacement, demonstrating a substantial increase in the degree of short-range order. To obtain further insight into the amorphous structure, Kolobov et al. [17] performed X-ray absorption near-edge structure simulations. The mechanism of the phase transition can be explained as follows. The Ge atoms play a key role in the amorphization procedure. They occupy the octahedral and tetrahedral symmetry positions in the crystalline and amorphous states, respectively. The difference is that several weaker covalent bonds connect the Ge atoms with other atoms. Upon application of an intense laser, the weaker bonds will be ruptured, and the Ge atom will flip into the tetrahedral position. By checking the Ge–Te distance, this explanation fits the experimental data well. They suggested that electronic excitation creating nonequilibrium charge carriers is crucial for weakening and subsequent rupture of the subsystem of weaker Ge–Te bonds. Photogenerated nonequilibrium carriers populate these states, making them more susceptible to thermal vibration-induced dissociation. The local arrangement around the Sb atoms remains unchanged, enhancing the overall stability. Both of these factors contribute to amorphization.

#### 2.1.2. Properties of GST and Other Chalcogen-Based Optical PCMs

GST is widely used and commercialized in materials that are used in optical data storage devices, such as digital videodisks (DVDs). Researchers are attempting to discover new properties while searching for new applications of the currently known properties of GST. By slightly changing GST and exploring its new properties, research has been extended from GST to some general chalcogen-based optical PCMs (O-PCMs).

**Figure 2:**
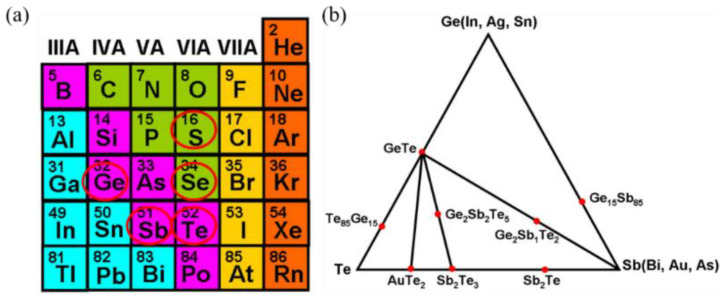
(**a**) Typical elements in the chalcogenide family used for optical storage. (**b**) The tertiary Ge-Sb-Te phase diagram with some popular chalcogenide alloys highlighted.

Chalcogen-based PCMs consist of some particular elements that are widely used for memory. The commonly used elements are shown in Figure 2a [18]. Other than these elements, researchers have also discovered other components that have potential phase-change properties; for example, AuTe_2_, which is also included in the chalcogen-based materials family.

The discovery of a new class of materials along the GeTe–Sb_2_Te_3_ pseudo-binary line brought about the initial use of GST alloys. The basic properties were known long before, and it was not until the emergence of the pseudo-binary line that GST alloys attracted much attention because of their properties. Alloys along this line with compositions (GeTe)*_m_*(Sb_2_Te_3_) have the ability to rapidly switch between the phases and have strong electrical/optical contrast [19]. After doping the material with elements (mostly those in Figure 2a, crystallization will change, which could result in an even faster switching speed or a steadier amorphous phase [20,21]. The GeTe–Sb_2_Te_3_ pseudo-binary line and the previously mentioned alloys are shown in Figure 2b [18].

**Figure 3:**
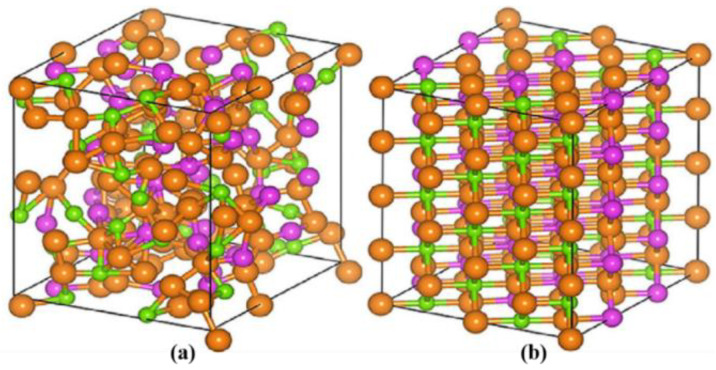
The structural phase transition of the GST chalcogenide PCM between its (**a**) amorphous and (**b**) crystalline states.

GST alloys and other chalcogen-based PCMs have similar optical properties that are beneficial for potential applications. They work by forward crystallization and backward amorphization, which is a way of phase transformation that is reversible. The procedure is shown in Figure 3 [13]. The two states of these materials show tremendous contrast of the optical constants. We mostly pay attention to the complex index of refraction, optical loss, and electrical resistivity [22]. These properties are fundamental to optical storage, light modulators, and other types of devices.

The phase-change process is based on the thermal effect. By applying a specifically designed laser pulse, the material will be heated above its crystallization temperature but below its melting point. After slow cooling, in contrast to amorphization, the atoms will show long-range order, so the material is in the crystalline state. If the material is heated above the melting point and then rapidly quenched to room temperature, it will reach the amorphous state. The two states have different electronic densities and transition matrix elements, that is, the oscillator strength of the optical transitions due to structural rearrangement. Another advantage of GST alloys and other chalcogen-based PCMs is that the phase-change processes of these materials can be very fast, and they are faster than those of almost all known materials.

Other properties are also important for this type of material to be used in devices. Requirements such as stability at room temperature, large scalability of the capacity, a nonvolatile nature, and extended read/write endurance are all fulfilled by GST alloys and other chalcogen-based PCMs, and they are necessary for applications.

In addition to the optical properties, the thermal properties should also be taken into consideration. The thermal conductivities of the different phases of Ge_2_Sb_2_Te_5_ range from 0.14 to 1.76 W/(m·K) [23]. The low thermal conductivity can significantly reduce energy consumption in the crystallization and amorphization procedure, which is beneficial for the optical properties.

### 2.2. VO_2_ and Other Transition-Metal Oxide O-PCMs

#### 2.2.1. Models and Physical Mechanism of the Phase Transitions of VO_2_

VO_2_ is one of the strongly correlated electron materials that are typically characterized by a variety of phase transitions that occur as a result of competing interactions between the charge, spin, orbital, and lattice degrees of freedom [24]. First-order phase transitions in solids, such as VO_2_, are notoriously challenging to study [25]. The transition-metal dioxides at the beginning of the d series have been studied by DFT-based electronic structure calculations, which are capable of correctly describing the electronic and magnetic properties of the metal, as well as both the first insulating monoclinic (M1) and the second insulating monoclinic (M2) phases. By using recently developed hybrid functionals, considerable progress has been made in understanding the physics of VO_2_, including the electron–electron interaction, and thereby improving the weaknesses of the semilocal exchange functionals provided by the local density and generalized gradient approximations [26]. Although there are limitations in the local density approximation, the theory establishes a reference that can be directly compared with experimental data [7].

**Figure 4:**
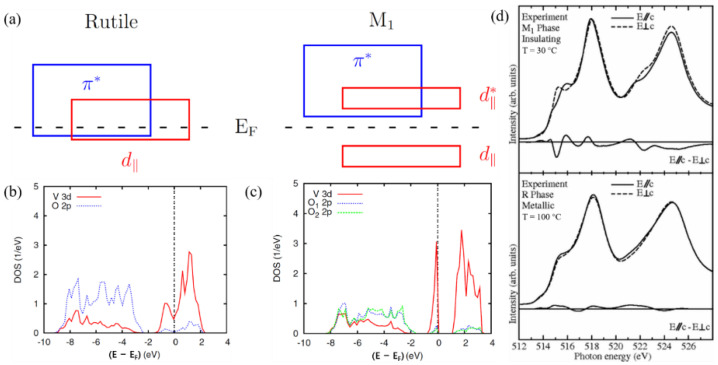
(**a**) Schematic band diagrams for VO2 in the metallic (rutile) phase and the semiconducting(monoclinic) phase. (**b**) Partial DOS of rutile VO2 as calculated using the HSE functional. (**c**) Partial DOS of M1-VO2 as calculated using the HSE (bottom) functional. (**d**) Experimental V L2;3 XAS spectra of VO2 in the insulating M1 phase (top panel, T = 30 °C) and the metallic R phase (bottom panel, T = 100 °C) taken with the light polarization $\vec{E}//c$ (solid lines) and $\;\vec{E}⊥c$ (dashed lines).

The results of calculations based on DFT using hybrid functionals are shown in Figure 4b,c, which are in good agreement with the experiment. The group of bands straddling the Fermi energy is dominated by the V_3d_ states. The V_3d_ and O_2p_ contributions in the energy range, where the respective other orbitals dominate, result from hybridization between these states. Therefore, the phase transition is accompanied by strong orbital switching, which has been confirmed by polarization-dependent X-ray-absorption spectroscopy measurements, as shown in Figure 4d. In addition, a constant critical free-electron concentration is needed on the insulating side to trigger the insulator-to-metal transition [24].

#### 2.2.2. Properties of VO_2_ and Other Transition-Metal Oxide O-PCMs

VO_2_ is a promising reconfigurable and reprogrammable active optical PCM because of its distinctive insulator-to-metal phase transition. It provides orders of magnitude change in the resistivity and large changes in absorption and the refractive index, which is accessible in the range from near- to far-infrared wavelength [27].

**Figure 5:**
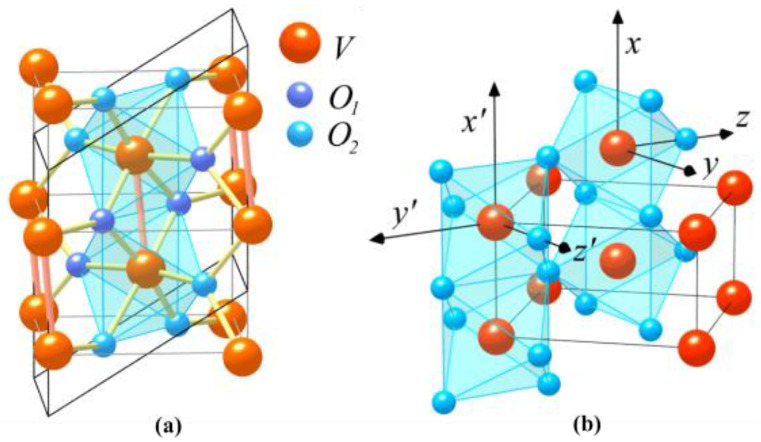
Crystal structures of (**a**) semiconducting(monoclinic) and (**b**) metallic (rutile) phases of VO_2_.

At a critical temperature of *T*_C_ = ≈67 °C (340 K), VO_2_ undergoes a significant and reversible insulator-to-metal phase transition in response to an increase in the temperature [28]. In the process of the phase transition, the structural transition occurs, with a change from a low-temperature monoclinic phase to a high-temperature rutile phase. The fundamental atomic structures of VO_2_ are shown in Figure 5 [7]. The insulator with a monoclinic structure is shown in Figure 5a, and its bandgap is about 0.7 eV. The metal with the rutile structure is shown in Figure 5b. The large and small spheres denote the metal and ligand atoms, respectively.

The phase transition of VO_2_ can also be induced by an applied terahertz electric field [29], hot-electron injection [30], strain [31], and all-optical pumping [32]. For all-optical pumping, the time of the phase transition is ~75 fs [33].

**Figure 6:**
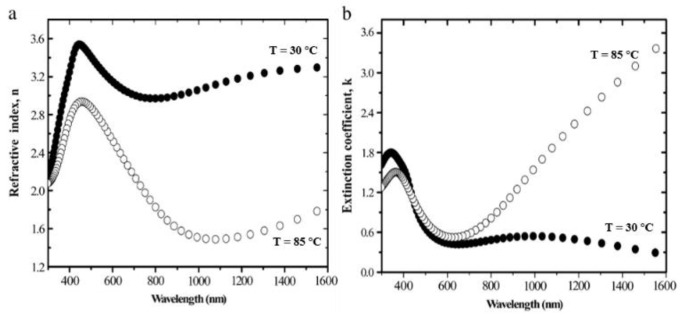
Wavelength dispersions of VO_2_ thin films. (**a**) Refractive index, n and (**b**) extinction coefficient, k. Thin films at temperatures below and above the phase transition temperature of 67 °C determined by UVISEL spectroscopic ellipsometry.

In the telecommunication wavelength bands near 1310 and 1550 nm, the changes of the real and imaginary parts of the refractive index of VO_2_ are obvious. At 1310 nm, the complex refractive index of VO_2_ changes from about 3.2 + 0.5i in the insulating phase to about 1.5 + 2.5i in the metallic phase. At 1550 nm, the complex refractive index of VO_2_ changes from about 2.9 + 0.4i in the insulating phase to about 2.0 + 3i in the metallic phase [34]. The wavelength dispersions of the refractive index (*n*) and extinction coefficient (*k*) of sputtered VO_2_ thin films in both states (semiconductor at *T* < *T*_C_ and metal at *T* > *T*_C_) are shown in Figure 6 [35].

There are very large changes in the optical, dielectric, and electrical properties of materials when the transition occurs with thermal excitation [36]. The transition from the dielectric to the metallic state can be as fast as femtoseconds (“on”), while the transition from the metallic state back to the dielectric state is slower, with transition times ranging from ~1–10 ps (“off”).

VO_2_ has been incorporated into silicon photonics, plasmonics, and other hybrid nanocomposites to achieve improved performance, and it also provides platforms for reconfigurable photonics and enables the construction of ultrafast optical switches, modulators, and memory elements [27]. However, VO_2_ is unsuitable for nonvolatile and reconfigurable applications.

## 3. Photonic Devices and Applications Based on PCMs

PICs provide a scalable hardware platform to solve the contradiction between the rate of energy efficiency improvement and the rapidly increasing computational load. PCMs have injected new life into on-chip PICs because of their programmability and wide use in reconfigurable photonic devices. In this section, we report the PCMs used in PICs, which contain an optical switch, an optical logical gate, and an optical modulator. Photonic neural networks based on PCMs are another interesting application.

### 3.1. Basic Concepts and Realization Principles

First, we introduce some basic concepts and nanostructures that are usually used in photonic devices. Fiber optics process parallel streams of data at high rates. The application of plastic optical fibers in communication has been limited to short data links and local area networks because the comparatively high attenuation and modest bandwidth of these fibers limit the transmission rate. However, these limits can be overcome by using a W-type fiber in which the waveguide dispersion is smaller than in the single-cladding fiber. The W-type fiber is also easier to splice. Moreover, the intermediate layer decreases the fiber’s effective numerical aperture, and therefore the number of guided modes, and confines the guided modes tighter to the core. As a consequence, it has a wide transmission bandwidth and lower bending losses compared with the corresponding single-cladding fiber [37]. The wide bandwidth is important for the computing speed.

Photonic crystals are artificial microstructures with a spatially periodic dielectric distribution. Because of the distinctive modulation actions of the periodic refractive-index distribution in space on the incident electromagnetic waves, a photonic bandgap (also called the stop band) is generated, which leads to the specific ability of a photonic crystal [38]. The refractive index is changed by a microstructured pattern of very small holes running along the length of the photonic crystal fibers. Generally, a photonic crystal fiber has a solid core and a holy cladding. The pattern of holes lowers the effective refractive index of the cladding, enabling the fiber to guide light. There are additional adjustable geometric parameters that provide greater design flexibility, so the bandwidth can be improved by tuning many parameters [39].

Microring resonators are compact bent dielectric waveguides in a small loop configuration. Controlling the signal light’s output can be realized by changing the coupling state between the waveguide and the microring through the control light. PCMs can change the index and other optical properties by controlling the phase transition, so optical routing and optical switching can be well realized.

Metamaterials refer to materials that are artificially structured to exhibit remarkable electromagnetic properties that cannot be found in nature. Metamaterials are structured on a sub-wavelength scale and can be described by an effective medium approach [4]. The use of metamaterials is the creation of arbitrary light field distributions on a sub-wavelength scale, and they can change the propagation direction, spatial amplitude, phase pattern, and polarization state of the incident light in some way. However, all of these concepts are limited to volatile applications. The reversibly switchable optical properties of PCMs enable active nonvolatile nanophotonics.

### 3.2. Photonic Devices and Applications

#### 3.2.1. Integrated Photonic On-Chip Devices

The explosive growth of global data has put forward an increasing demand on information processing technology for ultralarge data transmission capacity and ultrahigh information processing speed. PICs provide a scalable hardware platform to realize ultrahigh response speed, low photon power threshold, high conversion efficiency, and relatively low device cost. With the constant development in the PCM platform and integration of multiple materials platforms, more and more reconfigurable photonic devices and their dynamic regulation have been theoretically proposed and experimentally demonstrated, showing the great potential of PCMs in integrated photonic chips. In this part, we introduce the recent developments in PCMs and discuss their potential for integrated photonic on-chip devices.

All-optical switches with the properties of high on-chip performance, ultracompact structure, high speed, and low power are highly desirable as the essential building blocks of integrated photonics. An all-optical switch can be defined as a structure with a pump light controlling the ON/OFF transition of the signal light.

**Figure 7:**
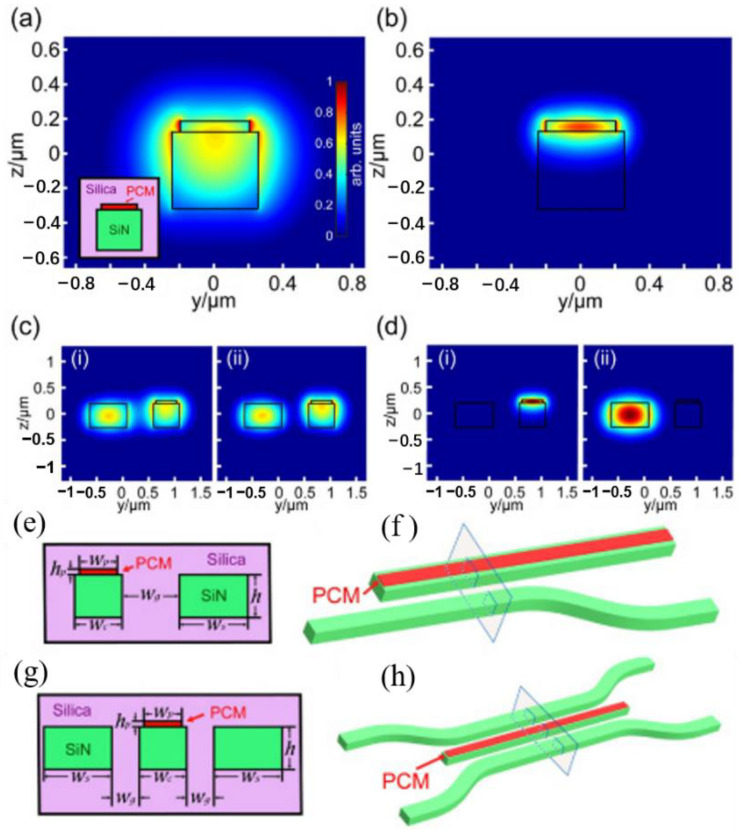
(**a**,**b**) Modal intensity profiles of a SiN waveguide loaded with a GSST strip in the (**a**) amorphous and (**b**) crystalline states. The inset in (**a**) illustrates the waveguide cross section; (**c**) intensity profiles of (i) even and (ii) odd supermodes in a two-waveguide system, one of which is topped with GSST, while the other is not. The GSST layer is in (**c**) the amorphous state and (**d**) the crystalline state. All modes are TE-polarized. (**e**) 1 × 2 switch: cross-sectional structure of the 1 × 2 switch. Here, Wc = Wg = 500 nm, Ws = 720 nm, Wp = 400 nm, h = 450 nm, and hp = 60 nm; (**f**) schematic illustration of the 1 × 2 switch, the rectangle marks the cross section depicted in (**e**). (**g**) 2 × 2 switch: cross section and (**h**) perspective view of the switch. Here, Wc = 512 nm, Ws = 730 nm, Wp = 400 nm, Wg = 562 nm, h = 450 nm, and hp = 60 nm. Wc: the width of the GSST-loaded silicon nitride (SiN) waveguide. Wg: the width of the gap between two waveguides. Ws: the width of the SiN waveguide without GSST. Wp: the width of the PCM. h: the height of the the SiN waveguide without GSST. hp: the height of the PCM.

In 2018, Zhang et al. [40] designed an O-PCM-based photonic switch that is broadband nonvolatile. They used a new O-PCM, Ge_2_Sb_2_Se_4_Te_1_ (GSST), to reduce the optical attenuation. The structure they designed is undisturbed, resulting in low-loss device operation beyond the classical figure-of-merit limit. The basic principle of this switching is to change the propagation states by phase transition. The electric-field intensities when GSST is in the amorphous (a-GSST) and crystalline (c-GSST) states are shown in Figure 7a,b, respectively. The even and odd supermodes in a two-waveguide system are shown in Figure 7c (i) and (ii), one of which is covered by a-GSST. The case of c-GSST is shown in Figure 7d. They designed a 1 × 2 switch (Figure 7e). When GSST is amorphous, the two waveguides are well coupled, so the light transfers into the other waveguide. When GSST is crystalline, the light will propagate without transfer. They also designed a 2 × 2 switch (Figure 7f), which works the same way.

**Figure 8:**
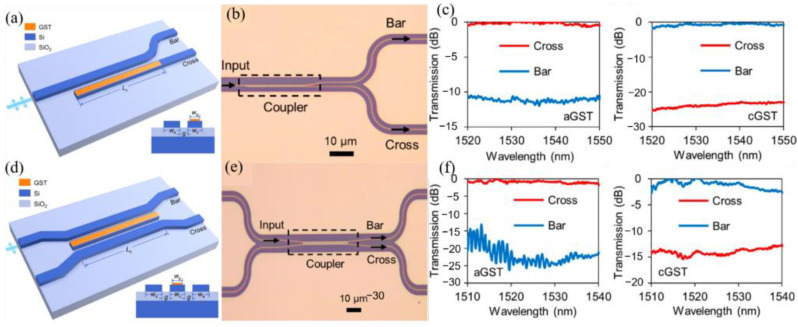
(**a**) Schematic of the design of the 1 × 2 DC switch. (**b**) Experimental results of the 1 × 2 DC switch, optical microscope image of the fabricated switch. (**c**) Measured transmission at the cross and bar ports with the GST in the amorphous and crystalline states. (**d**) Schematic of the design of the 2 × 2 DC switch. (**e**) Optical microscope image of the fabricated switch. (**f**) The measured transmission at the cross and bar ports with the GST in the amorphous and crystalline states.

In 2019, Xu et al. [41] reported a low-loss and broadband nonvolatile GST-based phase-change directional coupler (DC) switch. The basic principle is the same as that of Zhang et al. [40]. They demonstrated both a 1 × 2 DC switch (numerical, Figure 8a; experiment, Figure 8b) and a 2 × 2 DC switch (numerical, Figure 8d; experiment, Figure 8e). The experimental results are in good agreement with the numerical results.

**Figure 9:**
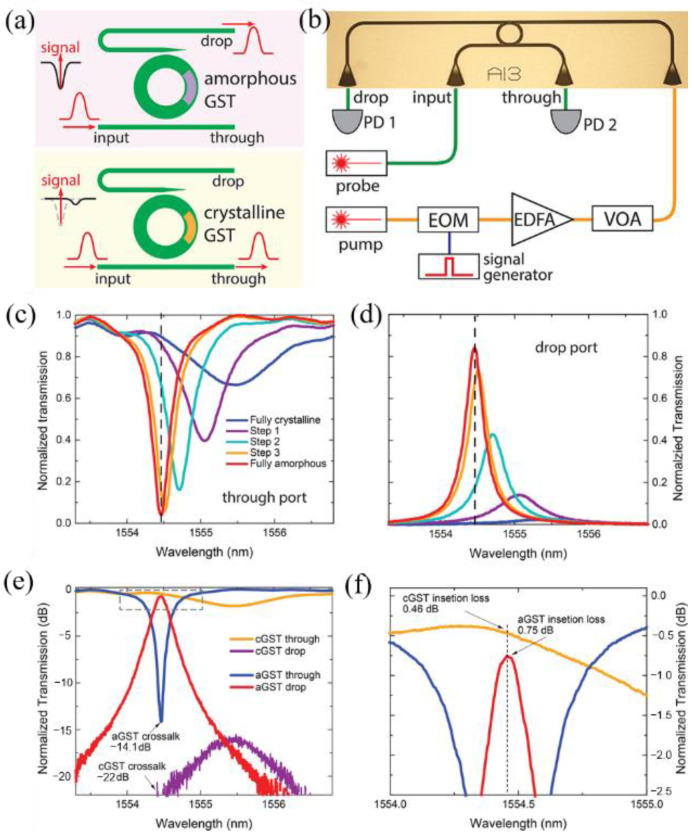
(**a**) The 1 × 2 optical switch operates by triggering the phase transition of the GST film embedded in a microring resonator. When the GST is in the amorphous phase, the signal resonantly couples into the microring and outputs from the drop port with a very low loss. When the GST is in the crystalline phase, the signal is decoupled from the microring and outputs directly from the through port to avoid any optical loss. (**b**) The measurement scheme uses pulses from a pump laser to optically control the phase transition of the GST, thereby switches the output of the probe laser between the through and drop ports. electro-optic modulator (EOM), erbium-doped fiber amplifier (EDFA), variable optical attenuator (VOA), photon detector (PD). (**c**) Through port and (**d**) drop port transmission spectra near a resonance of the microring when the GST is in a transition from the crystalline phase (blue) to the amorphous phase (red). (**e**,**f**) Log scale spectra highlighting the low insertion loss of 0.46 dB (0.75 dB) and crosstalk −14.1 dB (−22 dB) at the through (drop) port.

In 2019, Wu et al. [42] reported a PCM-based low-loss integrated photonic switch using GST as the PCM. Their system contains two waveguides and a microring. The microring is partly covered by GST. Schematic diagrams of the system are shown in Figure 9a,b. The through-port and the drop-port transmission are shown in Figure 9c–f.

**Figure 10:**
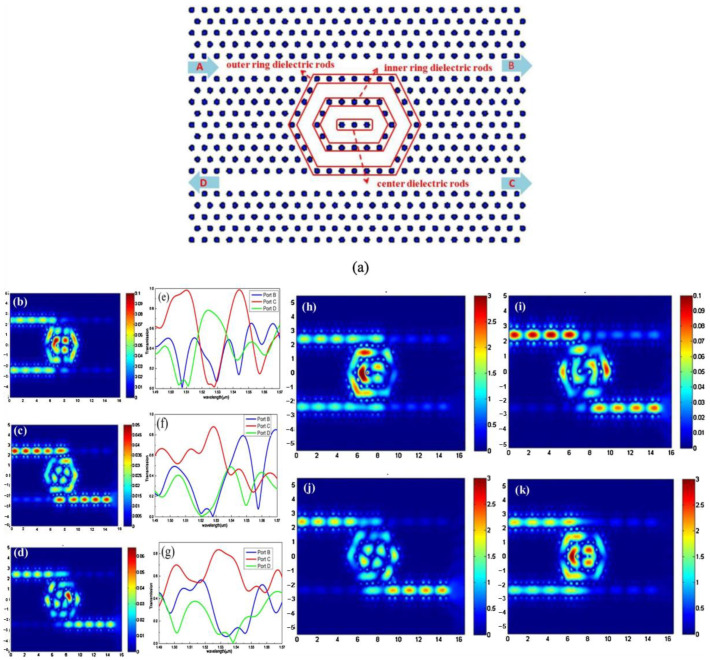
Schematic structure of the proposed PCM-based 2 × 2 PCDCS (**a**). The electric field distribution in the nested ring resonant cavity at 1530 nm (**b–d**) and its corresponding transmission spectra at ports B, C, and D (**e–g**) for the PCM-based 2 × 2 PCDCS with different refractive indexes of dielectric rods. (**b**,**e**) *n*_all_ = 3.6, (**c**,**f**) *n*_inner_ = 4.1 and *n*_other_ = 3.6, (**d**,**g**) *n*_all_ = 3.75. Electric field distribution at 1530 nm (**h**,**j**) and 1550 nm (**i**,**k**) when *n*_all_ = 3.6 (**h**,**i**) and *n*_inner_ = 3.95 and *n*_other_ = 3.6 (**j**,**k**).

In 2020, Zhang et al. [43] reported a PCM-based reconfigurable DC switch using a photonic crystal ring resonator. A schematic of the structure is shown in Figure 10. When the index of refraction is different, the port of the output light changes. They also realized double-wavelength switching, and the principle is the same. The 2 × 2 switch has five cross states, two dropping directions, works at two wavelengths, and only requires two materials.

Mimicking and implementing basic computing elements on photonic devices is one of the first and most essential steps toward all-optical computers. Programmable optical logic devices, such as logic “OR” and “NAND” gates, are the basic computing elements [44].

**Figure 11:**
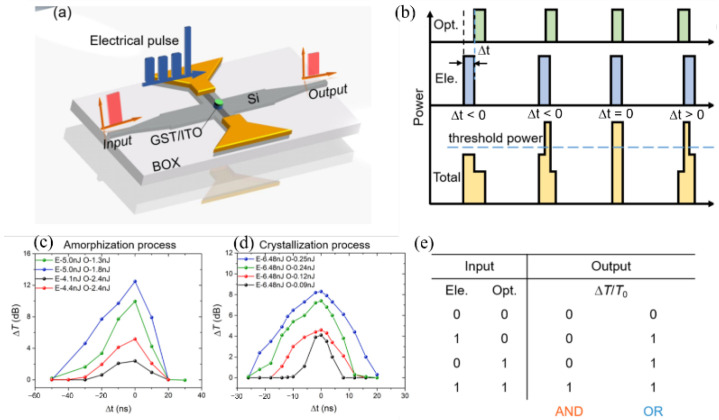
(**a**) Schematic of the GST-gated silicon multimode interferometer (MMI) crossing. (**b**) Co-interaction of optical pulses (green) and electrical pulses (blue) and the resulting power profile (orange). (**c**,**d**) Transmission change ∆T varies with the time difference between the two pulses ∆t in (**c**) amorphization and (**d**) crystallization processes. (**e**) The truth table of the “AND” and “OR” logic gates.

In 2019, Zhang et al. [45] reported a Si–Ge_2_Sb_2_Te_5_ hybrid photonic device induced by optical pulses for logic operation. The device structure is shown in Figure 11a, which is composed of a silicon multimode interferometer (MMI) crossing covered with a small circular piece of a 30 nm-thick GST layer and a 30 nm-thick ITO layer. A heavily P++ doped silicon strip orthogonally crosses at the center of the MMI with a Ti/Au layer deposited on top at the end of the doping region as an electrode, working as a resistive heater. The time delay between the electrical pulse and optical pulse affects the GST phase state and thus the response of the probe light, as shown in Figure 11b. Such a device can perform the Boolean logic operation. When the relative transmission change exceeds a certain threshold level, the logic output can be regarded as “1,” otherwise it is “0.” With low-energy pulses, the inputs “00,” “10,” and “01” give no or small transmission change, while the input “11” generates a very large change. If the threshold level is set as a 25% transmission change, it then accomplishes the “AND” logic operation. If the energy of the pulses is increased, only one pulse can significantly change the transition, exceeding the threshold. It then accomplishes the “OR” logic operation.

An optical modulator is one of the most important integrated optical devices in high-speed and short-distance optical communication. Optical modulation converts an electrical signal into an optical signal, and the modulated light wave is sent to the receiving end through the optical channel, where the optical receiver identifies its changes and then restates the original information. According to the modulation principle, optical modulators can be divided into electro-optic, thermo-optical, acousto-optic, and all-optical. When PCMs transform from the amorphous state to the crystalline state, their optical properties drastically change. Short optical or electrical pulses can be used to switch between these states, making PCMs fit in with the production of optical modulators. PCMs show enormous potential with respect to the device footprint, optical bandwidth, and extinction ratio because of their high contrast and broadband optical properties. Next, we report several optical modulators based on PCMs.

**Figure 12:**
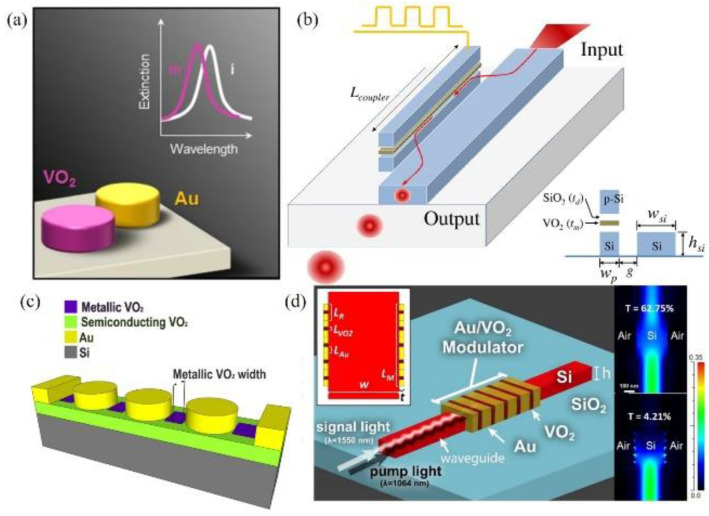
(**a**) Schematic of a phase-change nanomodulator based on Au and VO_2_ nanostructures. (**b**) Schematic view of the hybrid plasmonic modulator based on vanadium dioxide insulator-metal phase transition. The inset shows the cross-sectional view of the optical device. (**c**) Schematic illustrating regions of VO_2_ metallization when a voltage is applied across the gold nanodisk chain. (**d**) 3D waveguide structure, illustrating pump and signal light injection, poynting vector magnitude when VO2 is dielectric and metallic, for a 2D slab waveguide modulator.

In 2014, Appavoo and coauthor [46] proposed a type of modulator. The principle of the phase-change modulator based on Au and VO_2_ nanostructures is shown in Figure 12a. The VO_2_ nanostructure can adjust the resonance of the gold plasma structure in the device, thereby adjusting the characteristics of the hybrid modulator. Almost at the same time, using the demonstrated ultrafast dynamics of VO_2_ under optical excitation, a Si/VO_2_ hybrid ring resonator that can realize high-speed optical modulation with out-of-plane ultrafast optical excitation was reported [47]. The proposed device geometry is shown in Figure 12b, which can achieve an extinction ratio greater than 3 dB with ~1 dB insertion loss over the entire C-band (1.53–1.565 µm). In 2015, Markov et al. [48] reported a Si/VO_2_/Au electro-optic modulator based on the near-field plasmonic coupling that is expected to demonstrate ~9 dB/µm extinction ratio/length (Figure 12c). Additionally, the use of an Au/VO_2_ hybrid pattern on a silicon waveguide has been proposed for in-waveguide all-optical modulation [49]. A schematic of the modulator is shown in Figure 12d.

**Figure 13:**
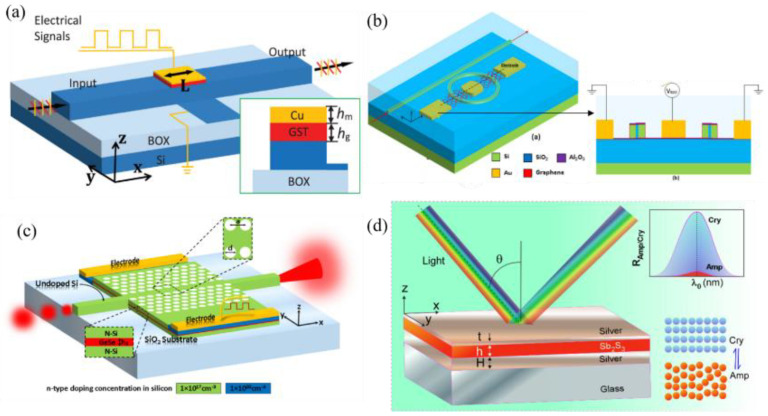
(**a**) Schematic of the proposed hybrid Si-GST-Cu waveguide modulator. The inset illustrated the cross-section view of the hybrid Si-GST-Cu waveguide. (**b**) Three-dimensional (3D) and two-dimensional (2D) schematic of the proposed electro-optic modulator. (**c**) Schematic of the proposed electro-optic modulator. Insets illustrate the cross-sectional views in different planes. (**d**) Schematic of proposed optical reflection modulator.

An ultracompact and ultralow energy consumption electro-optical modulator based on PCMs was reported by Yu and co-workers in 2018 [50]. By electrically triggering the phase of GST on the silicon waveguide, the absorption coefficient of the transverse electric mode switches. The structure of the GST-based compact electro-optical modulator is shown in Figure 13a, which is composed of a silicon bottom layer, a GST active middle layer, and a copper (Cu) top layer. In 2019, Ghosh et al. [51] reported a graphene-based electro-optic modulator in a slotted micro-ring resonator. Partially overlapping graphene layers, via graphene–Al_2_O_3_–graphene stack, were used above and below the slotted waveguide. A low-energy consumption electro-optic modulator based on the photonic bandgap shifting phenomenon in a PhC slab waveguide developed in a GeSe PCM layer has been reported [52], whose phase change from crystalline to amorphous, and vice versa, is by triggering electrical or optical pulses, thereby resulting in changes in the optical properties of the materials. The advantages of this modulator are its high extinction ratio. By exploiting the reversible and sub-nanosecond fast switching of antimony trisulfide (Sb_2_S_3_) from amorphous to crystalline, Chamoli et al. [53] reported a reflection modulator based on the metal–dielectric–metal structure in 2021. The advantages of this design are its small footprint, ease of fabrication, low cost, and high frequency of modulation.

The excellent properties of PCMs, containing fast and reversible switching of the states, considerable modulation in the electrical resistivity, and optical constants with a wide spectral region, greatly improve the performance of optical switches, as well as optical modulators in some aspects. The progress in photonic applications focusing on realizing reconfigurable and reprogrammable functions will give rise to new designs for an optical switch, optical modulator, and optical logic gate. The future possibilities of switchable dielectric and metallic nanostructures with PCMs are countless. Incorporating nonvolatile PCMs in integrated photonic devices enables indispensable reprogramming, reconfigurability, higher level of integration, and adjustability for integrated photonic on-chip devices.

#### 3.2.2. An Emerging Research Hotspot: Optical Convolutional Neural Networks

Artificial neural networks are already widely used in performing tasks such as face and speech recognition [54]. In more complex applications, such as medical diagnostics [55] and autonomous driving [56], fast data processing is more important.

Conventional digital computers usually adopt a von Neumann structure of operation, where computing architectures physically separate the core computing functions of memory and processing. The data are stored in the memory and executed in the central processing unit. As a result, fast, efficient, and low-energy computing is difficult to achieve. Computer architectures that can somehow fuse the two basic parts of processing and memory break through the bottleneck in terms of the overall speed of operation and amount of energy consumption. The neurosynaptic system consisting of neurons and synapses mimics the biological brain, and it is one of the computer architectures that can break through the limitation mentioned above.

**Figure 14:**
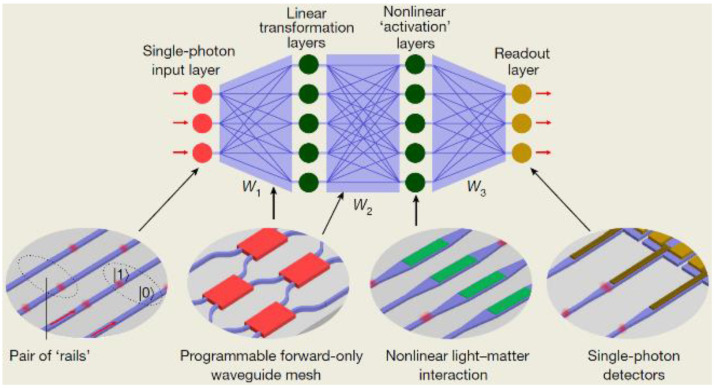
Quantum optical neural network based on programmable photonics. Such a network, implementing the matrix operations W1, W2, and W3, is fed by single photons and nonlinear activation (for example, nonlinear materials or atomic nonlinearities). The final state may be measured to complete a quantum computation or passed into a quantum network.

The computationally specific integrated photonic hardware accelerator (tensor core) is capable of operating at speeds of trillions of multiply-accumulate operations per second, which can meet the requirement of highly parallel, low-energy consumption, fast, and scalable hardware [57]. The tensor core is defined as the optical analogue of an integrated circuit that has a specific application, and it is always used in a neurosynaptic system or network. Photonic neurosynaptic networks promise access to the high speed and high bandwidth inherent to optical systems, thus enabling direct processing of optical telecommunication and visual data [58]. One way to implement quantum machine learning parallels classical photonic deep neural network accelerators, as shown in Figure 14. The stages of the linear waveguide meshes are connected by activation layers, but these activation layers must have strong coherent (reversible) nonlinearities. It is called a quantum optical neural network, and its task is training the phases in the waveguide mesh through supervised learning on the input and output quantum states [59]. Incorporating nonvolatile PCMs in integrated photonic devices enables indispensable reprogramming, reconfiguration, and in-memory computing capabilities for on-chip optical computing.

**Figure 15:**
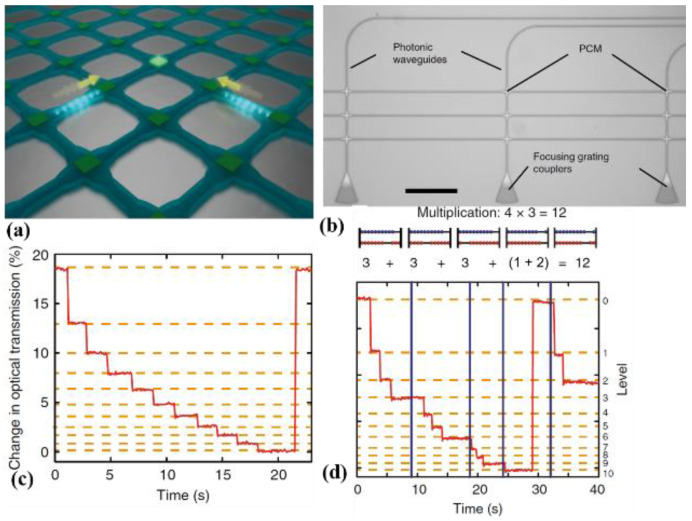
(**a**) Sketch of a waveguide crossing array illustrating the two-pulse addressing of individual phase-change cells. (**b**) Optical micrograph of a studied crossed-waveguide photonic array (Scale bar is 100 μm). (**c**) Level definition: the pulse energies are set in such a way that clearly distinguishable and repeatedly accessible levels are obtained. In this example, each step downwards consists of a group of five picosecond pulses, and the reset pulse of a group consists of ten ps pulses. (**d**) Multiplication: “4 × 3 = 12,” computed applying sequential addition.

For example, in 2017, Feldmann and co-workers [60] embedded nanoscale PCMs with a nanophotonic waveguide crossing array (Figure 15a), and selective addressing and manipulation with two overlapping pulses provides sufficient power to switch the desired PCM cell. The integrated central element for an all-optical calculator, a photonic abacus, was experimentally demonstrated, as shown in Figure 15b. This circuit operates at telecom C-band wavelengths (1530–1565 nm) with picosecond optical pulses. The single processing unit consists of a straight waveguide with a 7 μm-long PCM on top (Figure 15c). Transmission of the waveguide sensitively depends on the phase state of the PCM cell in a full switching cycle. Starting from the amorphized state, the PCM cell will be crystallized stepwise by identical picosecond pulses with pulse energies of 12 pJ. Every crystallization step needs five consecutive pulses, and the device is re-amorphized with ten 19 pJ pulses. With picosecond optical pulses, this system performs the fundamental arithmetic operations of addition, subtraction, multiplication, and division. For instance, they demonstrated base-10 multiplication of “4 × 3,” which can be implemented by successive addition (Figure 15d). As expected, the final states of the PCM cells after four successive pulse sequences and one carryover were “2” and “1,” thus representing the correct result of “12.” Each PCM cell represents a single place value (ones, tens, hundreds, and so forth) corresponding to the different rods of an abacus. The rectangular crossed waveguide array is scalable, and its size is limited by the absorption of the PCM cells. This research provides the first steps towards light-based non-von Neumann arithmetic.

**Figure 16:**
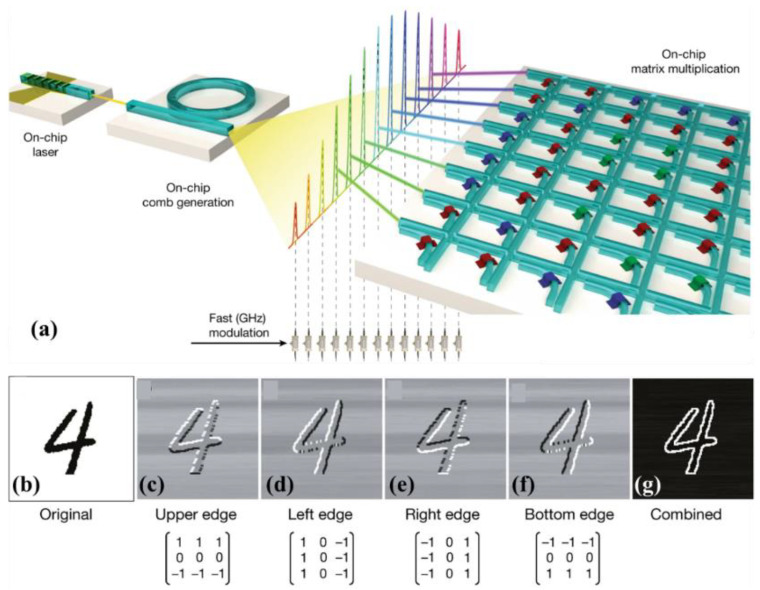
(**a**) Conceptual illustration of a fully integrated photonic architecture to compute convolutional operations. (**b–g**) Experimental result of convolving an image of 128 × 128 pixels showing a handwritten digit (**b**) with four image kernels of size 3 × 3 (corresponding to a 9 × 4 filter matrix). The kernels are chosen to highlight different edges of the input image. g, Combined image from c–f showing edge highlighting.

In 2021, Feldmann et al. [57] reported a tensor core that achieves parallelized photonic in-memory computing using PCM memory arrays and photonic chip-based optical frequency combs. The optical tensor core is scalable, has an ultralow loss, and is CMOS compatible. A conceptual illustration of the fully integrated photonic architecture is shown in Figure 16a, where an on-chip laser pumps an integrated Si_3_N_4_ microresonator to generate a broadband soliton frequency comb. The key is encoding of the image data onto the individual comb teeth of an on-chip frequency comb and subsequent encoding of the fixed convolutional kernels in the amorphous or crystalline phase of the integrated PCM cells that evanescently coupled to a matrix of interconnected photonic waveguides. The researchers experimentally demonstrated convolution using sequential matrix-vector multiplication operations (Figure 16b–g).

**Figure 17:**
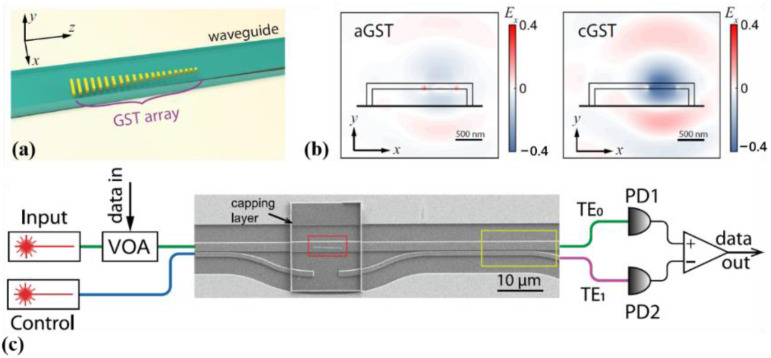
(**a**) 3D illustration of the devices (**b**) Finite Difference Time Domain (FDTD) simulation of the scattered electric field by one nano-antenna when the GST is in amorphous phase (left panel) and crystalline phase (right panel), respectively, showing the distinctive difference. (**c**) Scanning electron microscope (SEM) image of the complete device and the measurement and control schematics. The complete phase-change metasurface mode converter device consists of an encapsulated GST phase gradient metasurface (red box) and a mode selector (yellow box). the transverse electric fundamental mode (TE_0_), the first electric fundamental mode (TE_1_).

Similarly, in 2021, Wu and co-workers [1] proposed a multimode photonic computing core composed of an array of programmable mode converters based on on-waveguide metasurfaces made of the GST PCM. They then demonstrated a prototypical optical convolutional neural network that can perform image processing and recognition tasks with high accuracy, broad operation bandwidth, and a compact device footprint. A three-dimensional schematic diagram of the configuration is shown in Figure 17a, consisting of a linear array of GST nanoantennae integrated on a silicon nitride waveguide. Each GST nanoantenna scatters the waveguide mode and causes a phase shift Φ, which depends on its width and the refractive index of its material. The GST’s distinctive refractive index between the amorphous and crystalline phases can modify the scattered phase of each GST nanoantenna and control the conversion of the waveguide’s two spatial modes (modes TE0 and TE1), as shown in Figure 17b. The measurement and control scheme and a scanning electron microscope image of the complete phase-change metasurface mode converter device are shown in Figure 17c. With these advances and after overcoming the scaling challenge, the photonic neural network accelerator will be very promising for artificial intelligence in data centers where massive optical interconnects have already been deployed.

## 4. Conclusions

Optical PCMs are promising materials that can be used in reconfigurable and reprogrammable applications. They can also be incorporated into silicon photonics, plasmonics, and other hybrid nanocomposites to improve optical device performance. Two types of PCMs are widely used: chalcogenide compounds and metal oxides. One of the metal oxides is VO_2_, although it is unsuitable for nonvolatile and reconfigurable applications. Chalcogenide compounds, such as GST, have attracted more attention than other PCMs because of the high contrast in both the electrical and optical properties between the amorphous and crystalline states. The optical properties and the physical mechanism of the phase transition of these optical PCMs have been introduced in this review. In addition, the models and experimental data have been reported. Optical PCMs can be used in different micro/nano-optical structures, such as waveguides, microring resonators, surface plasmon polaritons, photonic crystals, and metamaterials, to realize various optical devices. The basic concepts have been introduced.

On-chip photonic devices, such as optical switches, optical modulators, and optical logic gates, are necessary to achieve PICs. The materials used in integrated optics are not suitable for active tunability. Optical PCMs have the potential to solve this problem, and they play an important role in improving the performance in terms of faster data processing speed, shorter response time, higher contrast, and lower energy consumption. The application of PCMs in reconfigurable and reprogrammable photonic devices has attracted extensive attention because of their excellent performance, and optical convolutional neural networks are also attracting great interest. Novel all-optical storage can realize high-density storage with multilevel storage [61], and it is useful to construct photonic neural networks. Integrated photonic hardware accelerators based on PCMs can break through the bottleneck in terms of the overall speed of operation and amount of energy consumption, as well as accelerate the development of future photonic circuits.

In conclusion, incorporating nonvolatile PCMs in integrated photonic devices enables indispensable reprogramming, reconfigurability, higher level of integration, adjustability, and in-memory computing capability for on-chip optical computing.

## 5. Discussion and Outlook

PCMs offer a versatile material platform for reconfigurable photonic devices because of their outstanding properties, such as fast and reversible switching of the states and drastic nonvolatile changes in the optical properties between the crystalline and amorphous states, accompanied by considerable modulation in the electrical resistivity and optical constants with a wide spectral region, across ultraviolet, visible, and infrared frequencies. During the last decade, PCM platforms have been developed for application in telecommunication, imaging, data storage and display technologies.

Limitations in the optical applications based on PCMs remain, as well as unsolved problems and technical problems that need to be solved. The limitations will be described in detail below, and we will propose some possible solutions.

First, in visible spectra, chalcogenide compounds simultaneously exhibit large extinction of both the refractive index and optical loss. The concurrent index and loss change fundamentally limit the scope of many optical applications. One possible solution is to discover new materials as the foundation of the research on optical PCMs. For example, a new class of optical PCMs based on GSST has been introduced because it can break the traditional coupling of both the large contrast of the refractive index and the large contrast of the optical loss [6]. Moreover, the researchers pointed out that isoelectronic substitution with light elements is a generic route in the search for new O-PCMs optimized for low-loss photonic applications.

Second, the mechanisms of the phase transitions in some PCMs are challenging to study, such as for VO_2_. The problem of metal–insulator transitions in transition-metal compounds has been a matter of ongoing controversy for a long time. The center of the problem is the relative role of the electron–lattice interactions and corresponding structural distortions versus the electron correlations [62]. The mechanisms of the phase transitions and models describing the metal–insulator transitions should be further investigated. A more appropriate method should be applied in the calculations, and then the theory will be in better agreement with the experimental data, such as X-ray absorption spectroscopy data.

The sub-wavelength structures of photonic devices based on PCMs are mostly fabricated by the following methods: ultraviolet (UV) photolithography, electron beam lithography (EBL), and gallium focused ion beam (FIB) milling [63]. The utilization of UV photolithography for nanoscale patterning is limited by the resolution, design and fabrication of the photomask, and diffraction effects. EBL can provide higher resolution and precision, but it might be incompatible with other nanofabrication processes, such as high-temperature chemical vapour deposition (CVD). Moreover, a tradeoff should be made between the unexpected phase transition caused by baking the photoresist at a high temperature and the resolution and accuracy of the pattern by baking at a lower temperature. FIB milling is a direct nanostructuring technique that is unconstrained by the limitations of the etchant and substrate compatibility, and it is incredibly versatile and compatible with a large variety of materials. However, proper calibration of the ion beam parameters is important to avoid redeposition of the milled material and gallium contamination. Different methods have their own advantages and disadvantages. Considering that there is no perfect fabrication method, the fabrication method should be carefully chosen. We look forward to the development of a better micro/nanofabrication method in the future.

The area of the devices using GST is usually large because they operate in the near- and mid-infrared frequency range, leading to poor integration. To reduce the size of optical devices based on PCMs, they should operate at a shorter wavelength. However, for photonic devices operating in the visible to near-infrared visible wavelengths, GST is not ideal owing to the relatively small change in its refractive index but large extinction coefficients, resulting in high losses [13]. A solution is to search for another PCM that is transparent at a shorter wavelength and possesses high optical contrast for the refractive index. The most promising candidates are GSST and Sb_2_S_3_. Sb_2_S_3_ has larger bandgaps for both the amorphous (*E*_g_ = 2.05 eV) and crystalline (*E*_g_ = 1.7 eV) phases than GST (*E*_g_ = 0.7 and 0.5 eV).

Finally, fully reconfigurable photonic devices are expected to have a large cycle number. Commercial PCRAM devices, which are based on GST, can be switched more than 10^8^ times, and the switching time can be as short as <500 ps. However, most of the photonics devices based on PCMs can only switch from amorphous to crystalline. As a result, the realization of such characteristics as those of commercial phase-change random access memory (PCRAM) devices within optical on-chip devices is still difficult [64]. This is because a fully reversible phase transition in PCMs via electrical and optical pulses needs to be performed with electrodes and protective layers, which influences the design and function of the optical devices.

Although some problems need to be solved, we believe that workable plans will be applied to break through the limitations of the applications of PCMs, and rapid progress will be made in photonic integrated circuits with ultrafast, ultrahigh bandwidth, and low-energy signaling. The reconfigurability will also give rise to new designs for smart glasses, displays, and reprogrammable devices.

The future possibilities of switchable dielectric and metallic nanostructures with PCMs are countless. Many structures with different functionalities, such as filters, lenses, absorbers, and sensors, can be imagined. With the rapid development in technological advances, a nascent technology platform and proliferation of phase-change-based nanophotonic devices into commercial device platforms will occur in the next few decades.
